# Case Study of How Alleviating “Pebbles in the Shoe” Improves Operations in the Emergency Department

**DOI:** 10.5811/westjem.24990

**Published:** 2025-03-24

**Authors:** Diana Savitzky, Yash Chavda, Suchismita Datta, Alexandra Reens, Elizabeth Conklin, Matthew Scott, Christopher Caspers

**Affiliations:** * NYU Langone Hospital—Long Island, Department of Emergency Medicine, Mineola, New York; † NYU Grossman Long Island School of Medicine, Department of Emergency Medicine, Mineola, New York

## Abstract

**Objectives:**

Addressing minor yet significant frustrations, or “pebbles,” in the workplace can reduce physician burnout, as noted by the American Medical Association. These “pebbles” are small workflow issues that are relatively easy to fix but can significantly improve the workday when resolved. This quality improvement project aimed to enhance clinician well-being in an emergency department (ED) affiliated with an academic institution through human-centered design by actively engaging clinicians to identify these “pebbles” and for a dedicated team to address them.

**Methods:**

A task force comprised of three emergency physicians collaborating with emergency medicine leadership was established. After educating clinicians about “pebbles,” clinicians were able to anonymously submit pebbles based on recall of frustrations in a baseline survey at the start of the project, as well as submit pebbles in real time by a QR code that was placed in easily noticeable areas. The task force met bimonthly to categorize, prioritize, and assign ownership of the pebbles. Progress was communicated to staff via a monthly “stop light” report. An anonymous survey assessed the impact on clinician well-being among 68 emergency clinicians within seven months of starting the project.

**Results:**

Over seven months, 284 pebbles were submitted (approximately 40 per month). The feasibility of addressing pebbles was characterized by a color scale: green (easy to fix): 149 (53%); yellow (more complex): 111 (39%); and red (not feasible, “boulder”): 24 (8%). Categories of pebbles included the following: equipment/supply: 115 (40%); nursing/clinical: 86 (30%); process: 64 (23%); and information technology/technology: 19 (7%). A total of 214 pebbles (75%) were completed. Among 51 respondents (75% response rate), the self-reported impact on well-being of having pebbles addressed was as follows: extremely effective: 16 (31%); very effective: 25 (49%); moderately effective: 8 (16%); slightly effective: 2 (4%); and not effective 0 (0%).

**Conclusion:**

In addition to improving personal resilience, improving well-being in the ED involves addressing efficiency of practice. This project highlights the positive impact of resolving small, feasible issues identified by clinicians, which resulted in 80% of respondents rating the project as very to extremely effective in improving their well-being. Most pebbles were related to equipment and easily fixed, while issues involving human interactions (eg, communications between consultants and EM) were more challenging. Regular meetings and accountability facilitated progress. This approach is replicable across medical specialties and practice settings, offering a low-cost method to enhance clinician work environments and well-being.

## INTRODUCTION

Physician burnout is one of the most discussed topics in emergency medicine (EM).^1^–^3^ It can be exacerbated by the buildup of minor workflow problems. Small yet significant issues can create substantial inefficiencies, workforce dissatisfaction, and potentially lead to medical errors.^4^ The American Medical Association mentioned in an editorial article that these “pebbles in the shoe” should be addressed to reduced burnout and promote organizational change.^5^,^6^ In one example, leaders at Wisconsin’s Marshfield Clinic Health System solved about 30% of their pebbles by systematically listening to team members and prioritizing issues based on feasibility and impact.

Much of a physician’s well-being at work is rooted in the work environment.^7^ Many studies have advocated for systems-level improvements, rather than just targeting the individual physician.^8^, ^9^ The Stanford Model of Professional Fulfillment incorporates three different key drivers of professional fulfillment: culture of wellness; efficiency of practice; and personal resilience.^10^ Culture of wellness involves leadership support, infrastructure, and resources to promote well-being. Efficiency of practice refers to systems that ensure quality, safety, and effectiveness, with physician involvement in redesigning inefficient processes being crucial.^11^ Solving “pebbles in shoe” addresses both the culture of wellness and efficiency of practice. Addressing minor problems quickly can minimize burnout and increase efficiency.^5^,^10^, ^11^

Our objective was to allow clinicians to identify their operational frustrations at work and convey this information to the ED leaders that could fix them and to show the impact this project had on clinician well-being.

## METHODS

### Setting and Participants

The study was conducted in an academic emergency department (ED) at a tertiary care center with over 80,000 annual visits. Participants included 68 clinicians (32 attending physicians, 6 emergency medicine residents, and 30 advanced practice practitioners [APP]).

### Intervention

A “pebbles task force” was formed, consisting of two lead emergency physicians; EM leadership, including the department chair; department administrator; nursing administrator; director of pediatric EM; and director of supply chain. The objective was to address minor workflow issues impacting clinician satisfaction. By streamlining information from frontline physicians and leadership, the process aimed to save time and create efficiency. A baseline pebbles survey was distributed to identify existing workplace frustrations, and a QR code system was implemented to collect real-time pebble submissions. QR codes labeled “real-time pebbles” were placed near computer workstations in easily noticeable areas so clinicians could easily scan them without having to search for them. Of note, clinicians had to scan the QR code on their own accord; they were not emailed or prompted to do so during their shift.

**Population Health Research Capsule**

What do we already know about this issue?

*Minor workflow frustrations—what we define as “pebbles”—contribute significantly to physician burnout and inefficiencies in the ED.*

What was the research question?

*We aimed to evaluate how systematically addressing these minor workflow issues could enhance clinician well-being in an ED setting and reduce physician burnout.*

What was the major finding of the study?

*Of 284 “pebbles” submitted, 75% were resolved. Most were related to equipment and supply (40%). 80% of clinicians rated this project as very to extremely effective.*

How does this improve population health?

*This approach can indirectly enhance clinician well-being and workforce retention while increasing efficiency.*

### Study of the Interventions

We collected pebbles using Qualtrics software (Qualtrics International Inc, Provo, UT). The baseline pebble survey asked participants to recall minor frustrations encountered while working clinically. A prospective pebble survey, used in real time via QR code was sent to all attendings, APPs, and residents. It included three questions: “What frustrated you just now?”; “What area of the ED are you working in?”; and “What is your role?.” Submissions were kept anonymous to allow for free expression of frustrations; however, there was a suggestion to include medical record numbers for clinical issues and/or the individual’s name if they wanted to be contacted about this issue, as well as a prompt to escalate the issue in real time to the nurse manager or supply team.

Pebbles were entered into a database (REDCap hosted at http://openredcap.nyumc.org/) and categorized at the discretion of the task force in two ways: by type (eg, equipment/supply, IT/technology) and area of the ED (eg, critical care, peds ED) and by perceived feasibility based on the initial submission (green = easy to fix, ≤3 months; yellow = can be fixed but more complex and would take >3 months; red = cannot be fixed). Key stakeholders from the task force were assigned responsibility for follow-up on each pebble based on suitability. For example, when clinicians mentioned needing otoscopes and ophthalmoscopes, the director of supply chain ordered and installed the equipment. For more complex issues, our department administrator or nursing manager would work with other departments or units but assume responsibility for reporting back to the task force on progress.

The pebbles task force met bimonthly to review submissions and prioritize based on feasibility and potential impact. Progress was communicated via a monthly “stop light” report, indicating the status of issues as green (completed), yellow (in process), or red (not feasible, “boulder”). Explanations for unfeasible items, such as regulatory issues preventing rapid strep swabs in certain areas, were also provided. “ were made to complete the pebbles within three months.

If a pebble was submitted multiple times, a full review was conducted to determine why the problem was recurring. For example, for repeat supply issues, a new initiative, “Inventory Chat,” was created to address broken or missing equipment through a messaging system on work iPhones, delivering real-time communication to inventory staff.

### Outcome Measures

The primary outcome measures included the number and characteristics of pebbles submitted, the feasibility of addressing each pebble, and the self-reported impact on clinician well-being. The predicted feasibility of each pebble was categorized as green (easy to fix within three months), yellow (more complex and take >3 months to fix), or red (not feasible, “boulder”). A secondary outcome measure of well-being impact was assessed via an emailed anonymous survey after seven months that simply asked clinicians, “How effective is the “Pebbles in Shoe” project in improving your personal sense of wellbeing?” on a five-point Likert Scale.

### Analysis

We conducted a quantitative analysis on the number and characteristics of pebble submissions, their completion rates, and feasibility. The impact on clinician well-being was assessed through qualitative analysis of responses from a single-question survey, focusing on the perceived effectiveness of the intervention in improving work conditions and reducing burnout.

## RESULTS

### Description of Pebble Submissions

Over the seven-month period, 284 pebbles were submitted, averaging approximately 40 submissions per month. The majority of pebbles, 74%, were submitted by physicians (residents and faculty), as compared to 26% by APPs. The distribution of types of pebbles across categories is shown in Table 1. The predicted feasibility of addressing the submitted pebbles is shown in Table 2, relative to all pebbles submitted.

**Figure 1:**
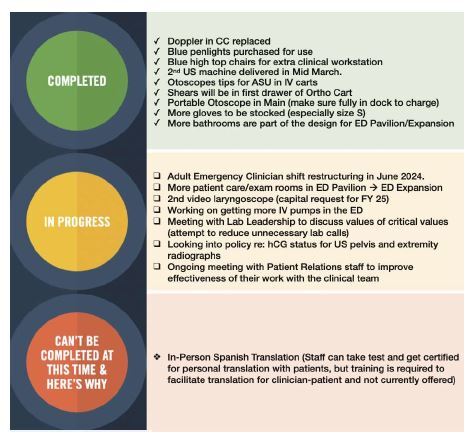
“Pebbles in shoe” project traffic stoplight report Winter Quarter 2024. *CC*, critical care; *ASU*, Ambulator Surgical Unit; *US*, ultrasound; IV, intravenous; *ED*, emergency department; *Ortho*, orthopedic; *FY*, fiscal year; *hCG*, human chorionic gonadotropin.

Overall, 214 pebbles (75%) were completed, indicating a high rate of feasible solutions within the identified issues. This high completion rate underscores the effectiveness of the task force in addressing and resolving minor workflow issues promptly. Figure 1 shows an example of a “traffic stoplight” report for the Winter Quarter that demonstrates the progress of the Pebbles Project to clinicians.

### Impact on Well-being

An anonymous survey with a 75% response rate showed that 80% of clinicians rated the project as very to extremely effective in improving well-being. (See Table 3 for details.) Additionally, 80% of clinicians rated the project as very or extremely effective in improving well-being.

## DISCUSSION

### Summary

This project highlights the positive impact of resolving small, feasible issues identified by clinicians, which resulted in 80% of respondents rating the project as very to extremely effective in improving their well-being. The task force successfully addressed many minor issues; the most frequent “green” pebbles were related to equipment and supply, which were generally easier to resolve. For instance, a frustrating manual stapler was replaced with an electric one. Human interaction issues, like nursing/clinical problems, were categorized as being “yellow pebbles” and were more challenging but addressed effectively by our nursing leadership. For example, one of the first successful pebbles involved COVID-19 nasal swabs. After multiple submissions, the task force collaborated to shift this responsibility from clinicians to nursing staff. Several submissions also led to a shift-restructuring pilot to address inequities in patient distributions for clinician teams. While it is important to address the “green,” easy-to-fix pebbles to keep clinicians engaged with the project by celebrating small wins, taking the time to fix the “yellow/red” pebbles was equally important, as they had a greater impact.

### Interpretation

This project demonstrates that systematic, clinician-driven identification and resolution of workflow issues, or “pebbles,” can enhance their clinical work environment and well-being. Engaging frontline staff gave clinicians a greater sense of autonomy, while collaboration between departmental leadership and ED stakeholders ensured success. The high completion rate underscores the effectiveness of the approach. Regular meetings and accountability were key to the project’s success. The project has become so successful that “pebble” has become a verb in the department.

### Comparison with Existing Literature

The findings align with existing literature emphasizing the importance of addressing everyday frustrations to reduce burnout. Previous literature has shown that reducing unnecessary stressors can significantly enhance job satisfaction and reduce turnover rates.^11^–^13^ At Marshfield Clinic, a similar “pebble” initiative resulted in a 30% completion rate, while our sustained efforts led to a 75% completion rate.^6^

## LIMITATIONS

This study was performed in a single center with a highly motivated team. Without dedicated individuals, replicating the project elsewhere may be challenging. Managing the databases, preparing reports, and tracking progress required significant effort. While the project fell within the existing budget, an estimated 0.1 FTE should be allocated to lead clinicians. While not a trivial cost, this financial investment has the potential to improve the work experience, thereby decreasing burnout and improving retention, as it is well known that replacing physicians is very costly. Reliance on self-reported data may introduce bias, and future studies should consider validated survey tools. Additionally, surveying well-being pre-/post-intervention may offer stronger evidence for this project to enhance clinician well-being. Expanding to other departments or multiple sites could help validate the findings.

## CONCLUSION

Regularly addressing small workflow issues through clinician feedback can enhance work environments and well-being. This method is low cost and replicable across various medical settings, providing a practical approach to reducing physician burnout. It is already being incorporated into other departments within our hospital as well as a nearby affiliated emergency department.

## Figures and Tables

**Table 1:** Distribution of pebble categories by frequency.

Pebble categories	Number of pebbles (%)
Equipment/Supply	115 (40%)
IT/Technology	19 (7%)
Nursing/Clinical	86 (30%)
Process	64 (23%)

*IT*, Information technology.

**Table 2:** Distribution of predicted pebble feasibility and progress of solving the problem.

Predicted feasibility of pebble problems	Number of ebbles (% of all pebbles)
Green (easy to fix)	149 (53%)
Yellow (more complex)	111 (39%)
Red (not feasible, “boulder”)	24 (8%)
